# Quality of life of adult individuals with intestinal stomas in Uganda: a cross sectional study

**DOI:** 10.4314/ahs.v21i1.53

**Published:** 2021-03

**Authors:** Yasin Ssewanyana, Badru Ssekitooleko, Bashir Suuna, Emmanuel Bua, Joseph Wadeya, Timothy K Makumbi, William Ocen, Kizito Omona

**Affiliations:** 1 Department of Surgery, College of Health Sciences, Makerere University, Kampala, Uganda. yasinsewa@gmail.com Tel; +256774988991; 2 Department of Surgery, Mulago National Referral and Teaching Hospital, Kampala, Uganda; 3 Faculty of Health Sciences, Uganda Martyrs University. komona@umu.ac.ug, Tel: +256706464873

**Keywords:** Stoma, Quality of Life (Stoma-QOL), Psychological effects, Patient Health Questionnaire (PHQ-9), Generalized Anxiety Questionnaire (GAD-7)

## Abstract

**Introduction:**

Intestinal stomas remain important life-saving surgical options in a wide range of gastrointestinal pathologies globally. Living with a stoma has potential to impair the patient's quality of life, often with associated negative psychological effects.

**Objective:**

To evaluate the quality of life among intestinal stoma patients under Mulago National Referral Hospital (MNRH), with emphasis on psychological effects and effects on family-social interactions.

**Methodology:**

A cross-sectional study carried out at surgical outpatient clinics of MNRH between January and June 2018. Data was collected using Stoma-QOL questionnaire, PHQ-9 and GAD-7 from 51 participants who had lived with intestinal stomas for at least a month.

**Results:**

Of the 51 participants, male: female ratio was 4:1 and aged 18–84 years (mean age 44.04+18.47 years). 76.5% had colostomy; 23.5% had ileostomy. Majority (88.2%) had temporary stomas. The overall mean Stoma-QOL score was 55.12+ 17.04. Only about a quarter (24%) of participants had Stoma-QOL scores >70 (best). Most patients exhibited negative psychological effects (anxiety-100%, concerns about changed body image - 96.1% and depression - 88.4%).

**Conclusion:**

Most participants had low levels of stoma-related quality of life, suffered negative psychological effects and exhibited limited social interactions. This calls for efforts to support Stoma patients adapt beter life.

## Introduction

### Background to the study

An intestinal stoma is a surgically created opening of the intestine onto the front wall of the abdomen, that allows removal of feces from the body, to drain into a pouch or other collection device^1^. The stoma can be temporary or permanent depending on its indication and may be put as part of an emergency procedure or planned electively. Over the past three centuries, intestinal stomas have significantly improved surgical outcomes for a wide range of pathologies, reducing both morbidity and mortality^2^,^3^.

The lack of sphincter function results in fecal and flatus incontinence. There is an abrupt transition from having no stoma to having one, sometimes with no prior knowledge of such likelihood.

Quality of life(QOL) is the individual's perception of their health status in relation to social, physical, psychological, economic and spiritual aspects^4^. The concept of QOL broadly encompasses how an individual measures “goodness” of multiple aspects of their life^5^. Research directed to studying QOL has become increasingly important since the 1960's^6^ and QOL is now a major factor to consider when instituting any form of therapy^7^. Several studies documented low levels of quality of life specifically because of having the stoma^8^–^15^.

Stoma patients have a myriad of sensitive issues to address that include, changed body image, loss of control over elimination of feces and flatus, managing the stoma and trying to continue with normal function among others^16^,^17^. A review of several publications found out that 25% of stoma patients experience depression, anxiety and negative emotions following stoma surgery and that 50% of patients had worries about their altered body image, 47% felt they had lost confidence, 23% felt sexually altered and unattractive^18^. Stomas have been reported as a restricting factor of social life and many of the patients get less active socially and feel restrictions in re-creation, transport, sports and leisure time activities^19^–^24^.

Although such negative effects of intestinal stomas on patients have been reported from a variety of settings, these have been only speculative with respect to Ugandan stoma patients.

### Broad objective

The study aimed to assess the quality of life of adult intestinal stomas patients under the care of Mulago National Referral Hospital, focusing on the psychological effects of stomas and their effects on family-social interactions of the affected individuals.

### Specific objectives

The study had the following specific objectives;

1. To determine the Overall Stoma-Quality of life (Stoma-QOL) in Mulago National Referral Hospital.
2. To establish the psychological effects of intestinal stomas on adult individuals with intestinal stomas in Mulago National Referral Hospital.
3. To determine the associations between patient characteristics and psychological effects of Intestinal Stomas in Mulago National Referral Hospital.
4. To find out the effects of Intestinal Stomas on Family-Social interactions of adult individuals with intestinal stomas in Mulago National Referral Hospital.

## Patients and methods

### Study Design

This was a descriptive and analytical cross-sectional study conducted in the general surgical outpatient clinics of Mulago National Referral Hospital between January, 2018 and June, 2018.

### Study Area

Mulago National Referral Hospital is the highest-level government hospital in Uganda, located on Mulago Hill in the northern part of the capital Kampala. The department of General Surgery at MNRH has eight units of which only three; [2A, 2B and 3C] conduct intestinal stoma surgery on adults. Postoperative reviews are conducted in outpatient clinics that run every Tuesday, Wednesday and Thursday where 2–4 stoma patients are reviewed every week.

### Population and Samples

The study included all adult patients 18 years and above with intestinal stomas for at least 1 month and excluded individuals with mental disabilities that could limit their capacity to respond to questions. A sample of 51 study participants were purposively selected.

### Data collection instruments and procedures

Data was collected by the principal investigator with the help of four trained research assistants. Data was collected using a standardized, interviewer-administered questionnaires; 1) the stoma-QOL (Stoma – Quality of Life), 2) Patient Health Questionnaire (PHQ-9) and Generalized Anxiety Questionnaire (GAD-7).

The Stoma-QOL was particularly designed to assess stoma-specific QOL with 20 items that focus on factors directly related to life with a stoma^25^, ^26^. Participants responded to each of the 20 items on a 4-point scale with numbers referring to; 1 = always, 2 = Some times, 3 = rarely & 4 = Not at all. The Stoma QOL score is calculated as a proportion of the total sum of a participant's responses and the highest possible sum (80) and multiplying the resultant value by 100. Thus stoma-QOL is on a linear scale ranging from zero (0) to 100. The sample's overall Stoma-related QOL is presented as the mean of the individual scores. Stoma-related QOL scores were classified as; best (>70), good (51 – 70), poor (31 – 50) and worst (≤ 30)^27^, ^28^. All the 20 items were responded to for the tool to be analysed.

On the other hand, PHQ-9, which is a validated screening tool was used to assess depression. It had an excellent internal reliability and test-retest reliability. In this study scores of 5, 10, 15, and 20 represented cut-off points for mild, moderate, moderately severe and severe depression, respectively^41^, ^42^.

GAD-7, which is a validated screening tool was used to assess anxiety. Even if anxiety and depression symptoms frequently co-existed, factor analysis described them as distinct dimensions^41^, ^42^. In this study, scores of 5, 10, and 15 represented cut-off points for mild, moderate and severe anxiety, respectively.

The tools were first translated from English to Luganda and later translated back to English to ensure that there was no distortion of the message. The final consensus English-Luganda translation was then administered to those participants who could not comprehend English language. The tool was also pretested on 5 patints (not part of our sample).

Cronbach's alpha is a measure used to assess the reliability, or internal consistency, of a set of scale or test items. Thus, reliability of any given measurement refers to the extent to which it is a consistent measure of a concept and Cronbach's alpha is one way of measuring the strength of that consistency (43). For this study, α coefficient for Stoma QOL, PHQ-9 and GAD-7 was 0.8, 0.8 and 0.65 respectively.

### Data entry, analysis and presentation

Data was coded with serial numbers and entered into electronic Epi Data Version 4.2.0.0 by double entry method, re-checked and exported to Stata Version 14.0 for analysis.

Descriptive statistics such as proportions for categorical and ordinal variables were displayed in tables and graphs, while means, standard deviations and medians were used for continuous variables.

Psychological effects (Anxiety or depression) of the stoma were categorized in this study as Anxiety-related concerns (Stoma-QOL items 1 through 5), Depression-related concerns (Stoma-QOL items 15, 19 and 20) and Body Image concerns (items 9, 11 and 14) according to the respective responses. The participants were then grouped into two categories, those with at least one symptom (Always, Sometimes or Rarely) relating to anxiety, depression, body image concerns and those without any symptoms (Not at all) relating to the psychological effects. Effects of stomas on family-social interactions was also analyzed in a similar way using Stoma-QOL items (13, 16, 17 and 18).

Bivariate analysis was used to determine the psychological impact of the stomas and the effects they impart on family-social interactions of patients using Fisher's Exact test. Logistic regression model was used for multivariate analysis. A p-value < 0.05 was considered significant. Psychological effects were categorized as having symptoms or not (coded as 1 and 0 respectively) relating to a particular effect. Confounding between independent variables was present if there was a difference of at least 10% between crude and adjusted coefficients of the logistic model.

## Results

### Participants characteristics

A total of 51 participants were recruited with a M: F = 4:1 and age range of 18 to 84 years old. The mean age was 44.04+ 18.47 years (Table 1)

**Table 1:** Baseline Characteristics of Participants

S. No	Characteristic	Frequency (n) & Percentage (%)
**01**	**Gender** ▪ Male ▪ Female	40 (78.4) 11 (21.6)
**02**	**Age (in years)** ▪ 18–30 ▪ 31–45 ▪ 46–60 ▪ ≥ 61	15 (29.4) 14 (27.5) 9 (17.6) 13 (25.5)
**03**	**Education level** ▪ Primary ▪ Ordinary secondary ▪ Higher secondary ▪ University level	24 (47.1) 16 (31.4) 4 (7.8) 7 (13.7)
**04**	**Marital status before** ▪ Single ▪ Married ▪ Divorced ▪ Widowed	14 (27.5) 33 (64.7) 2 (3.9) 2 (3.9)
**05**	**Marital status after** ▪ Single ▪ Married ▪ Divorced ▪ Widowed	14 (27.5) 30 (58.9) 4 (7.8) 3 (5.9)
**06**	**Initial diagnosis** ▪ Intestinal obstruction ▪ Penetrating abdominal trauma ▪ Aganglionic colon ▪ Colorectal Cancer ▪ Others	23 (45.1) 9 (17.6) 1 (2.0) 12 (23.5) 6 (11.8)
**07**	**Types of Stoma** ▪ Colostomy ▪ Ileostomy	39 (76.5) 12 (23.5)
**08**	**Intent of stoma** ▪ Permanent ▪ Temporary	6 (11.8) 45 (88.2)
**09**	**Duration in months** ▪ 1–6 ▪ 7–12 ▪ 13–18 ▪ >18	36 (70.59) 10 (19.61) 3 (5.88) 2(3.92)

**S. No**= Serial Number

Intestinal obstruction was the most common initial diagnosis (45.1%), mainly due to sigmoid volvulus) followed by colorectal cancer (23.5%). Stoma duration ranged from 1–42 months with a mean duration of 6.33 months.

Majority of participants (78.4%) were males. Most (88.2%) had stomas of temporary intent. Most patients (70.59%) had stomas for between 1 and 6 months.

### Overall Stoma-QOL

**Figure 1:**
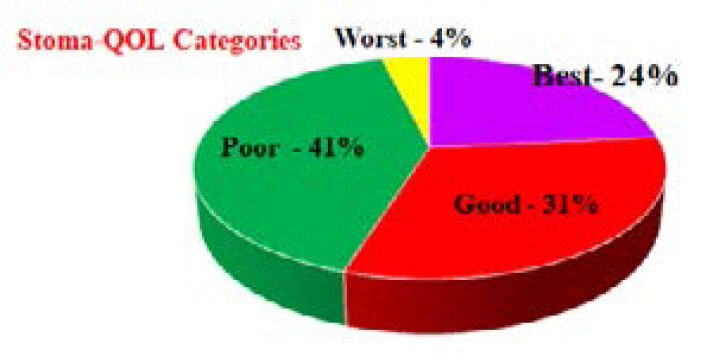
Classification of Participants' Stoma-QOL Scores

The mean Stoma-QOL was 55.12+17.04. The grouping of Stoma-QOL scores according to classification is shown in Figure 1 below.

Less than a quarter (24%) of participants felt that they had normal (“best”) Stoma-QOL with the remaining participants (76%) feeling that their QOL was sub-optimal (See figure 1).

### Psychological Effects of Intestinal Stomas on Adult Individuals with Intestinal Stomas in Mulago National Referral Hospital

Psychological effect can be of two major types; Anxiety or Depression. Thus, the psychological effects of intestinal stomas in this study were sub-categorized into; anxiety- related, concern about changed body image and depression-related effects. See table 2 below for details.

**Table 2:** Distribution of participant responses per item in the Stoma–QOL Questionnaire

Item	Always n (%)	Sometimes n (%)	Rarely n (%)	Not at all n (%)
1. I become anxious when the pouch is full	21 (41.2)	20 (39.2)	3 (5.9)	7 (13.7)
2. I worry that the pouch will loosen	22 (43.1)	13 (25.5)	6 (11.8)	10 (19.6)
3. I feel the need to know where the nearest toilet is	25 (49.0)	13 (25.5)	6 (11.5)	7 (13.7)
4. I worry that the pouch may smell	22 (43.1)	13 (25.5)	6 (11.8)	10 (19.6)
5. I worry about noises from the stoma	14 (27.5)	20 (39.2)	8 (15.7)	9 (17.6)
6. I need to rest during the day	10 (19.6)	17 (33.3)	14 (27.5)	10 (19.6)
7. My stoma pouch limits the choice of clothes that I wear	23 (45.1)	11 (21.6)	6 (11.8)	11 (21.6)
8. I feel tired during the day	7 (13.7)	18 (35.3)	9 (17.6)	17 (33.3)
9. My stoma makes me feel sexually unattractive	20 (39.2)	8 (15.7)	8 (15.7)	15 (29.4)
10. I sleep badly during the night	28 (54.9)	12 (23.5)	3 (5.9)	8 (15.7)
11. I feel embarrassed about my body because of my stoma	20 (39.2)	13 (25.5)	11 (21.6)	7 (13.7)
12. I worry that the pouch rustles	13 (25.5)	18 (35.3)	9 (17.6)	11 (21.6)
13. It would be difficult for me to stay away from home overnight	29 (56.9)	11 (21.6)	2 (3.9)	9 (17.6)
14. It is difficult to hide the fact that I wear a pouch	11 (21.6)	20 (39.2)	10 (19.6)	10 (19.6)
15. I worry that my condition is a burden to people close to me	25 (49.0)	10 (19.2)	3 (5.9)	13 (25.5)
16. I avoid close physical contact with my friends	21 (41.2)	11 (21.6)	7 (13.7)	12 (23.5)
17. My stoma makes it difficult for me to be with other people	17 (33.3)	18 (35.3)	8 (15.7)	8 (15.7)
18. I am afraid of meeting new people	20 (39.2)	12 (23.5)	6 (11.8)	13 (25.5)
19. I feel lonely even when I am with other people	10 (19.6)	21 (41.2)	5 (9.8)	15 (29.4)
20. I worry that my family feels awkward around me	7 (13.7)	12 (23.5)	8 (15.7)	24 (47.1)

**Anxiety;** All participants had concerns relating to anxiety at least rarely. Close to half the participants (41.2%) were anxious whenever the stoma pouches got full. Of all the participants, using Generalized Anxiety Questionnaire (GAD-7); Moderate to severe anxiety was seen in 12.36% and mild anxiety was present in 87.64%. Half of the respondents (49%) always felt the need to know where the nearest toilet was.

**Concerns About Changed Body Image;** Almost all participants (96.1%) had at least one concern related to body image. A significant proportion also felt of embarrassed with their new body image and felt sexually unattractive (Table 2).

**Depression;** Majority (88.24%) had depression-related concerns. Of these, based on Patient Health Questionnaire-9 (PHQ-9); moderate to severe depression – 37.35% and mild depression – 62.65%. Nearly half (49%) of participants always worried that they were a burden to the people close to them. Only less than half of the participants (47.1%) never had worries that their families felt awkward around them (Table 2).

### Associations between patient characteristics and psychological effects of intestinal stomas

To determine the association between patient characteristics and psychological effect of intestinal stomas, both univariate and bivariate analyses were done to show the distribution and the association respectively. See details in table 3 for univariate analysis and table 4 for bivariate below. Psychological effects were classified as; Depression, Changed Body Image and Anxiety.

**Table 3:** Univariate Descriptive analysis of participants characteristics versus psychological effects of intestinal stoma

Characteristic	Depression n (%)	Changed Body image n (%)	Anxiety n (%)
**Type of Stoma** ▪ Colostomy ▪ Ileostomy	34 (75.6) 11 (24.4)	38 (77.6) 11 (22.4)	35 (76.1) 11 (23.9)
**Intent** ▪ Permanent ▪ Temporary	4 (8.9) 41 (91.1)	4 (8.2) 45 (91.8)	5 (10.9) 41 (89.1)
**Gender** ▪ Male ▪ Female	36 (80) 9 (20)	39 (79.6) 10 (20.4)	38 (82.6) 8 (17.4)
**Educational level** ▪ Primary ▪ Ordinary secondary ▪ Higher secondary ▪ University	21 (46.7) 15 (33.3) 3 (6.7) 6 (13.3)	23 (46.9) 16 (32.7) 3 (6.1) 7 (14.3)	22 (47.8) 13 (28.3) 4 (8.7) 7 (15.2)
**Marital status before** ▪ Single ▪ Married ▪ Divorced ▪ Widowed	11 (24.4) 32 (71.1) 2 (4.4) 0 (0.0)	14 (28.6) 32 (65.3) 2 (4.1) 1 (2.0)	10 (21.7) 33 (71.7) 2 (4.3) 1 (2.2)

**Table 4:** Bivariate analysis of associations between patient characteristics and psychological effects of intestinal stomas

Characteristic	Depression p-values	Body image p-values	Anxiety p-values
**Type of stoma** ▪ Colostomy ▪ Ileostomy	0.565	0.419	0.665
Stoma Intent ▪ Permanent ▪ Temporary	0.141	**0.012** *	0.480
Gender ▪ Male ▪ Female	0.385	0.388	0.061
Education ▪ Primary ▪ Ordinary secondary ▪ Higher secondary ▪ University degree	0.540	0.169	0.629
Marital status before stoma ▪ Single ▪ Married ▪ Divorced ▪ Widowed	**0.004** **	0.152	**0.005** **
Age in years ▪ 18–30 ▪ 31–45 ▪ 46–60 ▪ ≤ 61	0.9767	0.1591	0.3664
Duration in Month ▪ 1–6 ▪ 7–12 ▪ 13–18 ▪ >18	0.4265	0.4785	0.2710

* There is association

** There is stronger association

Proportions of participants with concerns relating to depression, body image and social interactions were 88.24%, 96.1% and 90.2% respectively. Most of the participants who had colostomy were; Depressed-75.6%, had changed body image-77.6% and Anxious-76.1%. Psychological effects of Intestinal Stoma were more among the married participants; Depression-71.1%, Changed body image-65.3% and Anxiety-71.7%. Majority of the participants were male and thus Psychological effects were more felt among them; Depression-80%, Changed body image-79.6% and Anxiety-82.6% (see table 3 for details).

At bivariate analysis (table 4), Stoma intent was found to be associated with concerns about changed body image (p=0.012). Marital status before stoma was strongly associated with depression and anxiety (p=0.004 and p=0.005, respectively). However, there was no significant association for the other characteristics as shown in table 4 above.

### Effects of Intestinal Stomas on The Family-Social Interactions

Majority of participants (90.2%) had some limitation of social interactions that included difficulty in travelling, avoiding close physical contact even with close friends and fear to meet new people (See table 2). There was no statistically significant relationship between family-social interaction concerns and participant characteristic even though widowed individuals appeared twice more likely to have concerns when compared to other marital statuses at multivariate analysis (OR=2.0).

## Discussion

### Participant characteristics

This study had a male: female ratio approximately 4:1, a finding that does not differ much from those of several other studies conducted on intestinal stoma patients across the globe^13^, ^28^–^30^. The male predominance in our study could be partly because of the prevalent sigmoid volvulus in the central part of Uganda that is also predominantly a disease of males^31^. This also emerged as the commonest diagnosis leading to stoma formation in this study.

The participants had a mean age of 44.04 +18.47 years that is truly representative of the generally young Ugandan population (WHO, 2016). Relatively younger populations were reported in India^32^ and Egypt^30^.

The ratio of colostomy to ileostomy was high (3.25:1), a finding that is in agreement with those of several other studies from a wide range of settings^29^, ^30^, ^32^, ^33^. In the case of our setting, such a ratio is likely because large bowel disease is more prevalent than small bowel disease, as indicated by the initial diagnoses of the participants (Table 1). Ahmad and Khan in 2011, however, had a strikingly different distribution with 82% ileostomy and only 18% colostomy, owing to the high incidence of ileal perforations of infectious etiology in the setting of their study.

### Overall Stoma-QOL

Generally, less than a quarter of participants (24%) had normal Stoma-QOL scores. The mean Stoma-QOL score of our participants was the lowest among studies that used the same assessment tool. Several other studies however also had mean values generally below 60^28^, ^33^–^35^. We expected our study population to have significantly lower mean Stoma-QOL score than tho from other seemingly more developed settings, which was not the case. This is possibly because QOL is a subjective measure and contextualized at individual level. It could also be that the strong family support that most Ugandan patients get owing to the extended family structure observed in many parts of the country^36^ may have a compensatory effect with regard to psychosocial adaptation to the stomas.

There was a very big range of stoma-QOL values possibly due to the wide inequalities among the patients in terms of income, literacy levels, family support and the varying individual capacities in handling the stress associated with a stoma. The etiology of the low Stoma-QOL scores is most likely multifactorial but delineating these factors was beyond the aim of this study.

### Psychological Effects of Intestinal Stomas

Participants reported serious negative psychological effects in terms of anxiety, worries about changed body image and depression. To each of the concerns related to anxiety and body image, large proportions of the participants answered “Always”, indicating a general poor adaptation to stomas among the participants. This is not a surprising finding given the fact that there are no documented efforts in Uganda directed at helping these patients adapt to life with a stoma. A review of several studies reported that 25% of stoma patients experience depression, anxiety and negative emotions following stoma surgery and that half of patients had worries about altered body image and 23% felt sexually altered and unattractive^18^. Our study shows bigger proportions of patients suffering each of the mentioned negative psychological effects. These patients are thus in dire need of psychological support to overcome these challenges and attain better quality of life.

Almost half of participants (47.1%) said they never worry that their families feel awkward around them. This finding is promising as it indicates that a significant proportion of our patients still have a strong sense of belonging to their families and have high levels of hope, which has been correlated with better treatment outcomes^13^, ^37^. This suggests that little improvements in psychosocial support are likely to cause significant improvement in the stoma-related quality of life for these individuals.

### Associations between patient characteristics and psychological effects of intestinal stomas

No statistically significant relationship existed between most of the participant characteristics and the psychological effects or limitation of social interaction. A study in Cuba had however reported that ileostomy patients suffered less psychological effects when compared to their counterparts with colostomy^39^. Silva and colleagues also found no statistically significant difference in psychological issues between patients with temporary and permanent stomas^40^.

### Effects of intestinal stomas on family-social interactions

The participants had marked limitation of social interactions with over half of them always fearing to travel. Majority also had difficulty being with other people and feared meeting new people. All these indicate a greatly impaired social functioning. Related findings were reported from earlier studies conducted on intestinal stoma patients in a variety of settings^22^–^24^, ^38^. This impaired social functioning has implied negative consequences to the economic performance of these individuals. The extent of such effects can be evaluated by another study.

## Limitations of the Study

This study was a single-center study with a relatively small sample size. This negatively affects the external validity of the study and the results may not be used to give the national picture of the status of intestinal stoma patients in Uganda.

Having been conducted at a national referral hospital, this study was prone to selection (specifically; referral) bias and yet this was not controlled for.

It is possible that several other factors like income levels, stoma pouch quality, cultural beliefs, and religion among others could have influenced results of this study. These were not controlled for.

## Conclusion

Adult intestinal stoma patients under Mulago Hospital generally have sub-normal stoma-related QOL. They suffer serious negative psychological effects and significant limitation of social functions. There were no statistically significant relationships between these effects and most participant demographic characteristics even though widowed individuals appeared to be twice more likely to have anxiety concerns when compared to other marital statuses.

## Recommendations

The authors do recommend as follows;

1. There is need for a multicenter study with a larger sample size to help show the national picture of quality of life of intestinal stoma patients in Uganda.
2. There should be reorientation of the medical staff on comprehensive stoma care and ensure that psychotherapy (especially counseling) is started early in the postoperative period and continued during follow-up visits to enable patients adapt adequately to life with a stoma.
3. Intestinal stoma patients should be organized and encouraged to form peer groups so that patients can support each other to adapt better to life with a stoma through experience sharing.
